# An Adaptive and Secure Holographic Image Watermarking Scheme

**DOI:** 10.3390/e21050460

**Published:** 2019-05-02

**Authors:** Chuying Yu, Xiaowei Li, Xinan Chen, Jianzhong Li

**Affiliations:** 1 School of Physics and Electronic Engineering, Hanshan Normal University, Chaozhou 521041, China; 2 School of Electronics and Information Engineering, Sichuan University, Chengdu 610065, China; 3 Network and Educational Technology Center, Hanshan Normal University, Chaozhou 521041, China; 4 College of Mathematics and Statistics, Hanshan Normal University, Chaozhou 521041, China

**Keywords:** image watermarking, encrypted computer-generated hologram, entropy, sharp frequency localized contourlet transform, Schur decomposition

## Abstract

A novel adaptive secure holographic image watermarking method in the sharp frequency localized contourlet transform (SFLCT) domain is presented. Based upon the sine logistic modulation map and the logistic map, we develop an encrypted binary computer-generated hologram technique to fabricate a hologram of a watermark first. Owing to the enormous key space of the encrypted hologram, the security of the image watermarking system is increased. Then the hologram watermark is embedded into the SFLCT coefficients with Schur decomposition. To obtain better imperceptibility and robustness, the entropy and the edge entropy are utilized to select the suitable watermark embedding positions adaptively. Compared with other watermarking schemes, the suggested method provides a better performance with respect to both imperceptibility and robustness. Experiments show that our watermarking scheme for images is not only is secure and invisible, but also has a stronger robustness against different kinds of attack.

## 1. Introduction

Owing to the rapid growth of internet and multimedia technologies, the acquisition, transmission and exchange of digital multimedia data including images, audio and videos has become a simple task. On the other hand, digital images can be manipulated or reproduced easily by the use of powerful image processing tools. How to effectively protect the copyright of the digital products has become a significant topic. A great many techniques have been proposed for protecting the digital rights of image content recently. Among these methods, digital watermarking is viewed as the most promising solution for digital copyright protection. Digital watermarking is a process that hides a piece of secret information (watermark) in the original digital multimedia data for the purpose of copyright protection and its verification [1]. For the requirements of actual application, a watermarking system has some an essential characteristic, more specifically for its imperceptibility, robustness, security, and so on [2].

In recent years, a large number of image watermarking methods have been reported [2,3,4,5,6], which can be categorized into two groups: transform-domain and spatial-domain. In spatial domain schemes, the watermark is embedded directly into the original image by manipulating the pixel intensity values [3]. In contrast, the transform domain method inserts the watermark by changing the frequency coefficients of the original image in a transform domain. There are numerous transform domain watermarking techniques such as discrete wavelet transform (DWT) [4], discrete cosine transform (DCT) [2], fractional Fourier transform [5], gyrator transform [6] and singular value decomposition (SVD) [2], etc. As a powerful matrix decomposition technique, SVD has been widely applied in image watermarking [2]. To improve robustness, some hybrid SVD-based watermarking methods have been designed [2,4,7,8]. In comparison to spatial domain methods, these techniques are more robust against different image attacks. 

In general, robustness and imperceptibility, which are the most two important properties, are adopted to evaluate the performance of a watermarking system [9]. But these two characteristics oppose each other [9]. To achieve a tradeoff between transparency and robustness, adaptive watermarking algorithms [7,9] have been studied extensively recently. In recent years, entropy has been used to select adaptively the embedding positions which determine the performance of the watermarking scheme [2,4,10]. In [10], an image watermarking approach is proposed to insert the watermark into the DWT sub-band with the highest entropy. The major drawback of this method is that the quality of the watermarked image is seriously degraded. Since the above method is non-blind, the original image is needed in the watermark extraction process. Based on SVD and DCT, Lai designed a watermarking method where the watermark is superimposed on the blocks selected by entropy and edge entropy [2]. But this method cannot resist filtering, JPEG compression, blurring and rescaling attacks. To achieve a level of higher imperceptibility and robustness, Makbol et al. developed a block-based watermarking scheme based on SVD and DWT [4]. However, it is weak with respect to filtering, JPEG compression and blurring attacks too. 

Because of the encryption characteristic and the strong anti-interference property of a hologram, a new technique wherein digital holograms are employed as watermarks has been explored to increase the watermarking system’s performance [11,12,13] in recent years. In [11], based on the improved fuzzy c-means clustering and the iterative algorithm for embedding processes, an adaptive watermarking scheme that inserts the mark hologram into the DWT-transformed original image is proposed. The drawback of this method is high computation cost. Reference [12] reported that a hologram watermark was performed in the DWT domain by using an encrypted kinoform as watermark. The encrypted kinoform was generated by a non-cascade phase retrieval algorithm. The main shortcomings of this method are that the quality of the reconstruction of the mark kinoform is decreased and the phase retrieval algorithm has a high complexity. In previous work [13], the phase-shifting interferometry-based CGH was inserted into the contourlet-transformed host image, and the embedding parameter is optimized by the use of particle swarm optimization. However, the computational complexity of this optimal scheme is high. In the above-mentioned methods, the watermarks are gray-level holograms. Due the advantage of being stored, transferred and replicated [14], the binary hologram is superimposed on the low-frequency wavelet coefficients of the original image using quantization index modulation [15]. But the parameter of this watermarking method is determined experimentally. Additionally, the watermarking method with an adaptive texturized algorithm is also developed to protect digital hologram recently [16].

Though DWT has been applied widely in watermarking and image processing [8] due to its good properties such as multiscale and time frequency localization, it cannot capture the directional information of images effectively. This weakness is overcome by contourlet transform (CT) [17]. However, the efficiency of contourlets in representing smooth contours of an image is decreased because of some waste components [7,8]. This drawback is settled by use of sharp frequency localized contourlet transform (SFLCT) [18]. In addition, SVD alone is not preferred owing to the large computation cost [4]. Compared with SVD, Schur decomposition has the advantage of lower computational complexity because it is a major intermediate step in SVD [19,20]. Therefore, in this study, the advantages of SFLCT and Schur decomposition are considered to enhance the performance of image watermarking. Moreover, to gain both imperceptibility and robustness in terms of the watermarking requirements, the entropy and edge entropy are utilized to choose the suitable positions to embed the watermark. Furthermore, the security risks of the traditional holographic watermarking methods are increased because of the small key space of hologram watermark. Hence, to enhance the security of watermarking system, the chaotic maps that are extreme sensitive to initial conditions are adopted to develop a new encrypted computer-generated hologram algorithm in this work. The hologram fabricated by this algorithm has a huge key space. 

In this paper, a secure adaptive holographic watermarking scheme is presented. To enhance the security of the watermarking system, a novel encrypted binary CGH method based on sine logistic modulation map (SLMM) and logistic map is designed to fabricate a hologram watermark. The mark CGH is superposed on the original image which is transformed by SFLCT and Schur decomposition. The entropy and the edge entropy are employed to choose the positions which are suitable for embedding to achieve a high imperceptibility without losing the robustness in the embedding procedure. The watermark can be extracted without the host image during the watermark extraction process. Compared with other published schemes, the proposed method offers better performance better performance in both imperceptibility and robustness. Experiments exhibit that the proposed watermarking method, in addition to high security and transparency, has good robustness against different kinds of attack.

## 2. Related Background

### 2.1. Entropy and the Edge Entropy

To acquire the satisfactory level imperceptibility and robustness, different techniques are utilized to find optimal watermarking parameters [9]. Entropy has also been used extensively to support data-hiding algorithms. For an image, disturbances are much less visible in highly textured regions than in uniform areas, and the entropy can be used to describing the texture of it. The edge is regarded as another important image characteristic. But the edge points are not the suitable site for watermark embedding. Therefore, the edge entropy is an important factor that determines an image block to be selected for embedding whether or not. The entropy and the edge entropy were utilized to determine the embedding positions in the cover data so as to cause minimal perceptual distortion.

The entropy of an n-state system can be represented as follows [21]:

(1) $$ETP=-\sum_{i=1}^{n}p_{i}\log p_{i}.$$

where *p_i_* indicates the probability of occurrence of the event "*i*" with 0 ≤ pi ≤ 1 and $\sum_{i=1}^{n}p_{i}=1$.

The edge entropy can be described as follows [21].

(2) $$ETPE=\sum_{i=1}^{n}p_{i}\exp(\mu_{i})=\sum_{i=1}^{n}p_{i}\exp(1-p_{i}).$$

where *μ_i_* = 1− *p_i_* is the ignorance or uncertainty of the pixel value.

### 2.2. Chaos Functions

In this work, two chaos functions including the Sine Logistic modulation map (SLMM) and logistic map have been chosen to heighten the security of the CGH. 2D-SLMM is defined as [22]

(3) $$\left\{ \begin{array}{l} X(n+1)=\alpha (\sin(\pi Y(n))+\beta )X(n)(1-X(n))\\ Y(n+1)=\alpha (\sin(\pi X(n+1))+\beta )Y(n)(1-Y(n)) \end{array}\right.,$$

where 0 ≤ *α* ≤ 1 and 0 ≤ *β* ≤ 3 are control parameters. When parameter *β* is close to 3, SLMM works in a chaotic state [22].

The logistic map is a 1D chaos function and defined as [23]

(4) x(n+1)=γx(n)(1−x(n)),

where *γ* is the logistic map parameter, and *γ* ∈ [0,4], *x_n_* ∈ (0,1). When 3.5699456 < *γ* ≤ 4, logistic map exhibits chaotic performance [23].

### 2.3. Schur Decomposition

Suppose the size of an image matrix *A* is *N* × *N*, the Schur decomposition of *A* is defined as [19]

(5) $$A=USU^{T},$$

where *S* is the block upper triangular matrix and *U* is a unitary matrix. *U^T^* denotes the conjugate transpose of *U*.

## 3. Encrypted Binary Computer-Generated Hologram Based on Chaos

In this section, the chaotic maps are used to enhance the security level of a CGH. First, based on SLMM and logistic map, a scrambling algorithm is designed. Then the encrypted CGH method is developed by using Burch’s coding method and this proposed scrambling algorithm.

### 3.1. The Image Permutation Method Using SLMM and Logistic Map

Assuming that the size of the input image *I*(*x*,*y*) is *M* × *N*, the scrambling method is described as the following steps:

(1) Initialize *SX*(1), *SY*(1) and *SZ* which are between 0 and 1 randomly and choose an arbitrary natural number *T* first. Then with *SX*(1) and *SY*(1), iteratively generate two chaotic sequences *SX*(*i*) and *SY*(*i*) using Equation (3). The lengths of *SX*(*i*) and *SY*(*i*) both are *MN* + *T*. Here, *i* = 1, 2, …, *MN* + *T*.
(2) Generate two random integers *t*1 and *t*2 between 1 and *MN* + *T*. Then calculate the initial value *XL*(1) of logistic map according to the following Equation (6)
  (6) XL(1)={|[SX(t1)+SY(t2)]/2|−SZ}/10,
  where *SX*(*t*1) and *SY*(*t*2) are the *t*1^th^ element in *SX* and the *t*2^th^ element in *SY*, respectively.
(3) Using *XL*(1) and Equation (4), generate the chaotic sequences *XL*(*i*) whose length is *MN* + *T* iteratively. Here, *i* = 1, 2, …, *MN* + *T*.
(4) Generate a random integer *t*3 between 1 and *T*. Truncate *NM* elements of *XL*(*i*) from the *t*3^th^ element to obtain a chaotic sequence *SE* = {*XL*(*i*), *i* = *t*3, *t*3 + 1,…, *t*3 + *MN* − 1}.
(5) Subsequently, a new sequence *SP* and its corresponding permutation indices *ISP* can be obtained by sorting the sequences *SE* in ascending order. There are *MN* elements in *ISP*. The relations between *SE* and *SP* is *SP*=*SE*(*ISP*). For example, the *m*^th^ element in *SP* corresponds to the *ISP*(*m*)^th^ element in *SE*.
(6) Map *I*(*x*,*y*) into a 1D array *IZ* by use of the zigzag algorithm [24]. The length of *I*1 is *MN*.
(7) Then the permutation indices *ISP* is utilized to permute *IZ* and the scrambled vector *IV* can be achieved as follows
  (7) IV=IZ(ISP).
(8) Finally, the permuted image *SEI* can be achieved by applying the inverse zigzag scan process [24] to *IV*.

The inverse image permutation process is similar to the image permutation process. In inverse scrambling process, as described in steps (1)–(5), the permutation indices *ISP* is achieved first using the same initial values and control parameters of the chaotic functions. Then the permuted image *SEI* is mapped into a 1D vector *SEI*1 by employing the zigzag algorithm. Subsequently, permute *SEI*1 back to their original position according to the following equation

(8) DSI(ISP)=SEI1.

Finally, apply the inverse zigzag algorithm to *DSI* to retrieve the decrypted image *DI*. The parameters *SX*(1), *SY*(1), *SZ*, *α*, *β*, *γ*, *t*1, *t*2 and *t*3 are employed as private (secret) keys.

### 3.2. Encrypted Binary CGH

The encrypted CGH is generated as follows:

(1) In order to decrease the dynamic range of the hologram, a random phase *ψ*(*x*_0_,*y*_0_) which is uniformly in the interval [0,1] is multiplied to the image *f*(*x*_0_,*y*_0_) first.

(9) $$f_{1}(x_{0},y_{0})=f(x_{0},y_{0})\exp[j2\pi \psi (x_{0},y_{0})],$$

(2) Apply the Fourier transform to *f*_1_(*x*_0_,*y*_0_) to get the object wave *OW*(*x*,*y*).

(10) $$OW(x,y)=FT[f_{1}(x_{0},y_{0})]=A(x,y)\exp[j\varphi (x,y)],$$

where FT() is the Fourier transform operator. The amplitude and phase of *OW*(*x*,*y*) are *A*(*x*,*y*) and *φ* (*x*,*y*), respectively.

(3) Assume that the parallel reference wave is mathematically represented by function *RW*(*x*,*y*) = *A_r_*exp[*j*2π*ρφ_r_*(*x*,*y*)]. Here, *ρ* is the carrier frequency. The amplitude and phase of *RW*(*x*,*y*) are *A_r_*(*x*,*y*) and *φ_r_*(*x*,*y*), respectively. Sequentially, permute *OW*(*x*,*y*) and *RW*(*x*,*y*) to obtain the scrambled *O_s_*(*x*,*y*) and *R_s_*(*x*,*y*) by use of the proposed chaos-based permutation method shown in Section 3.1 with the parameters *SX*(1), *SY*(1), *SZ*, *α*, *β*, *γ*, *t*1, *t*2 and *t*3.

(4) The shuffled hologram transmittance *h*(*x*,*y*) can be achieved according to the following formula.

(11) $$\begin{array}{l} h(x,y)=|O_{s}(x,y)+R_{s}(x,y){|}^{2}\\ \;=|A_{s}(x,y){|}^{2}+A_{sr}^{2}+2A_{sr}A{\left(x,y\right)}_{s}\cos[2\pi \rho \varphi_{sr}(x,y)-\varphi_{s}(x,y)]\\ \;=C+2A_{sr}A_{s}(x,y)\cos[2\pi \rho \varphi_{sr}(x,y)-\varphi_{s}(x,y)], \end{array}$$

In Equation (11), let |*A_s_*(*x*,*y*)|^2^ + *A_sr_*^2^ be a constant *C*. *φ*_s_(*x*,*y*) and *A_s_*(*x*,*y*) are the phase and amplitude of *O_s_*(*x*,*y*), *φ_sr_* (*x*,*y*) and *A_sr_*(*x*,*y*) are the phase and amplitude of *R_s_*(*x*,*y*), respectively.

(5) In the light of Burch’s coding method, let |*A_s_*(*x*,*y*)|_max_ = 1 and *A_sr_* = 1, then *h*(*x*,*y*) becomes

(12) $$h(x,y)=0.5\{1+A_{s}(x,y)\cos[2\pi \rho \varphi_{sr}(x,y)-\varphi_{s}(x,y)]\}.$$

(6) Finally, fabricate the encrypted binary CGH *EBH* (*x*,*y*) by quantizing *h*(*x*,*y*), which is achieved by use of Equation (12), in 1-bit using OTSU algorithm [25].

The security of this encryption system is enhanced greatly because of the huge key space which is formed by the private keys including *SX*(1), *SY*(1), *SZ*, *α*, *β*, *γ*, *t*1, *t*2 and *t*3.

The reconstruction process of an encrypted binary CGH is described as follows:

(1) With the parameters *SX*(1), *SY*(1), *SZ*, *α*, *β*, *γ*, *t*1, *t*2 and *t*3, the encrypted CGH *EBH*(*x*,*y*) is scrambled by the proposed inverse permutation process mentioned in Section 3.1 to obtain *DEBH*(*x*,*y*).

(2) With the conjugate reference wave, the binarized reconstruction *RH* of the hologram can be achieved via utilizing inverse Fourier transform and OTSU algorithm. To improve the quality of reconstruction, the high-pass filter approach is employed to attenuate the DC item in the reconstructed image.

## 4. The Proposed Watermarking Method

### 4.1. Selection of Embedding Positions

As described in Section 2.1, to maintain imperceptibility and robustness to attacks, the entropy and the edge entropy are employed to select the embedding positions adaptively in our method. In addition, to strengthen the robustness, the watermark signal will be superimposed on the low frequency sub-band of the SFLCT-transformed original image in this work. The detailed steps that select the suitable blocks for watermark embedding are shown as follows.

(1) Divide the low frequency sub-bands of the SFLCT-transformed original image into non-overlapping blocks with *z* × *z* pixels first. Then compute the entropy and the edge entropy of each block by use of Equations (1) and (2), respectively.

(2) Sum up the two measure of entropy of each block according to the following equation.

(13) $$ETPS_{i}=ETP_{i}+ETPE_{i},$$

where *ETP_i_* and *ETPE_i_* are the entropy and the edge entropy of the *i*^th^ block.

(3) Sort the values *ETPS_i_* in an ascending order. Literatures state that the block with low *ETPS* value is suitable for embedding [2,21]. Thus, the block with smallest *ETPS* value is chosen for embedding the watermark signal until the number of selected blocks is equal to the number of watermark bits.

### 4.2. Watermark Embedding Algorithm

Suppose that *H* and *W* are the host image and the original binary watermark image, respectively. And their sizes are *M* × *N* and *P* × *Q*. Steps of embedding watermark into the original image are described as follows:

(1) Using the method described in Section 3, generate the encrypted binary hologram *CW* of the original watermark *W* first. Then map *CW* into a 1-D array *WM* by use of the zigzag scan process.

(2) Decompose *H* with 1-level SFLCT to achieve the low frequency sub-band *SL*.

(3) *SL* is divided into non-overlapping blocks of *z × z* pixels.

(4) In term of the Section 4.1, select *PQ* blocks which are suitable for embedding watermark signal.

(5) Apply Schur decomposition to all selected blocks.

(6) During the embedding process, an element of *WM* is superposed on one block. The (1,1)^th^ element in *S* matrix of the chosen block is altered to insert the watermark. To embed *WM*, the first element of *WM* is inserted into the first selected block, and then the embedding procedure is repeated until the rest elements of *WM* are inserted into the other chosen blocks in sequence. Let *S_i_* be the *S* matrix of the *i*^th^ selected block after Schur decomposition, *WM_i_* be the *i*^th^ element of *WM*. Here, *i* = 1, 2, …, *PQ*. The watermark is embedded via Equation (14).

(14) $$S_{i}^{\prime }(1,1)=\left\{ \begin{array}{l} S_{i}(1,1)-\Delta +0.25q,\;WM_{i}=0\;\mathrm{and}\;\Delta \in [0,0.75q)\;\\ S_{i}(1,1)-\Delta +1.25q,\;WM_{i}=0\;\mathrm{and}\;\Delta \in [0.75q,q)\\ S_{i}(1,1)-\Delta -0.25q,\;WM_{i}=1\;\mathrm{and}\;\Delta \in [0,0.25q)\\ S_{i}(1,1)-\Delta +0.75q,\;WM_{i}=1\;\mathrm{and}\;\Delta \in [0.25q,q) \end{array}\right.$$

where Δ = mod(*S_i_*(1,1),*q*), *S_i_*(1,1) is the (1,1)^th^ element in *S_i_* matrix, *q* is the quantization step. mod() is the modulo operation. Experiments show that the proposed method has high imperceptibility and good robustness against attacks when *q* ∈ [40, 60].

(7) Apply inverse Schur decomposition to every embedded block. Then the watermarked image can be obtained by use of inverse SFLCT. The *x*-coordinates and the *y*-coordinates of the first pixels of the selected blocks are saved in a 2 × *PQ* matrix. They will be used for watermark extraction.

### 4.3. Watermark Extraction

(1) First, the watermarked image is decomposed by 1-level SFLCT to achieve the low frequency sub-band *SL’*. Then *SL’* is splitted into non-overlapping blocks of *z × z* pixels.

(2) Using the stored the *x*-coordinates and the *y*-coordinates, all the embedded blocks can be obtained.

(3) Apply Schur decomposition to all obtained blocks.

(4) The watermark signal *WM_i_’* can be extracted by use of the following formula.

(15) $$WM_{i}^{\prime }=\left\{ \begin{array}{l} 0,\;\mathrm{if}\;\mathrm{mod}[S_{i}^{\prime }(1,1),q]<0.5q\\ 1,\;\mathrm{otherwise} \end{array}\right.,$$

where *S_i_’* be the *S* matrix of the *i*^th^ obtained block after Schur decomposition,

(5) By utilizing the inverse zigzag algorithm, the hologram watermark *CW’* can be achieved.

(6) With the private keys *SX*(1), *SY*(1), *SZ*, *α*, *β*, *γ*, *t*1, *t*2 and *t*3, the extracted hologram *CW’* is reconstructed in term of reconstruction process in Section 3.2.

**Figure 1:**
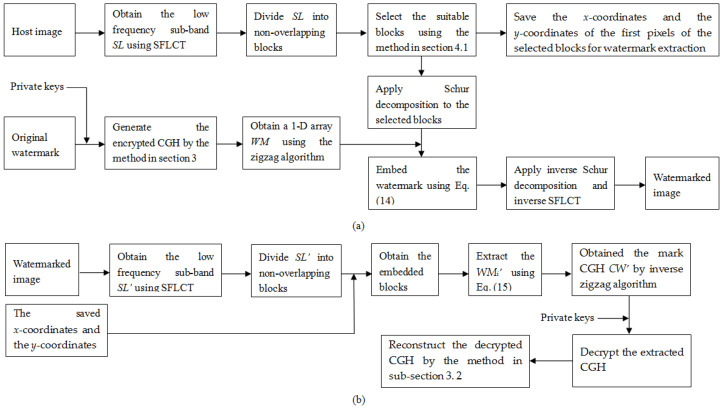
Flowchart of proposed watermarking method. (**a**) watermark embedding process; (**b**)watermark extraction process.

Figure 1 delineates the flowchart of the proposed method. In Figure 1a,b are the diagrams of the watermark embedding process and the watermark extraction process, respectively.

## 5. Experiments and Results

A set of experiments were performed to validate the proposed watermarking method using MATLAB.

### 5.1. Fabrication of the Encrypted Hologram

**Figure 2:**
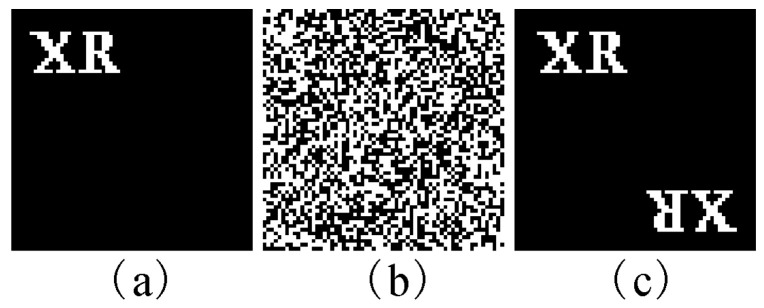
Results of the encrypted binary CGH. (**a**) the original image; (**b**) the encrypted binary CGH; (**c**) the binarized reconstruction of (**b**).

In the experiment, the encrypted binary CGH was fabricated by using the approach described in Section 3. Figure 2 depicts the original watermark and its corresponding reconstruction. The image, whose size is 64 × 64, in Figure 2a was employed to generate the encrypted hologram. Here, *SX*(1) = 0.352, *SY*(1) = 0.865, *SZ* = 0.752, *α* = 0.998, *β* = 3, *γ* = 4, *T* = 10^4^, *t*1 = 3528, *t*2 = 7832 and *t*3 = 6832, respectively. Figure 2b is the encrypted binary CGH. Figure 2c shows the reconstruction of Figure 2b with the all correct keys.

### 5.2. Test for the Effectiveness of Our Watermarking Scheme

A series of experiments were carried out to evaluate the imperceptibility and the robustness, which are two main requirements of the watermarking system according to the proposed method. The peak signal to noise ratio (PSNR) [26] was employed to measure the quality of the watermarked images. The watermarked image is within the acceptable degradation level if *PSNR* is larger than 30dB. Another metric normalized correlation (NC) [26], which is used to estimate the similarity between the original watermark and the extracted watermark, is utilized to evaluate the correctness of the extracted watermark. *NC* ∈ [0, 1]. Usually, it can be considered acceptable if *NC* is greater than 0.7. Higher NC value indicates good quality of extracted watermark. Bit error rate (BER) [27] is employed to calculate the difference between the reconstructed images of the embedded and extracted holograms. *BER* ∈ [0, 1]. The smaller the BER is, the better reconstructed image quality is. Experiments demonstrate the reconstructions obtained from the extracted watermarks cannot be recognized when their *BER* values are larger than 0.3. Ideally, *NC* = 1 and *BER* = 0.

**Figure 3:**
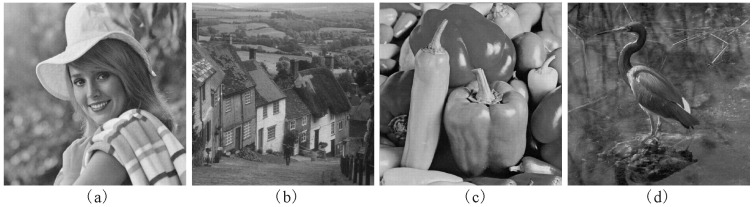
The host grayscale images. (**a**) Elaine; (**b**)Goldhill; (**c**) Peppers; (**d**) Crane.

The encrypted CGH in Figure 2b was used as the watermark in the tests. Four 1024 × 1024 grayscale images Elaine, Goldhill, Peppers and Crane given in Figure 3 were the host images. The quantization step *q* is 45 and *z* = 8. In terms of imperceptibility and robustness, the PSNR, NC and BER of our scheme are compared those of three other adaptive methods in [2,4,7]. For a fair comparison, the encrypted binary CGH in Figure 2b was adopted as the watermark in these four algorithms.

#### 5.2.1. Test Results for Imperceptibility

**Figure 4:**
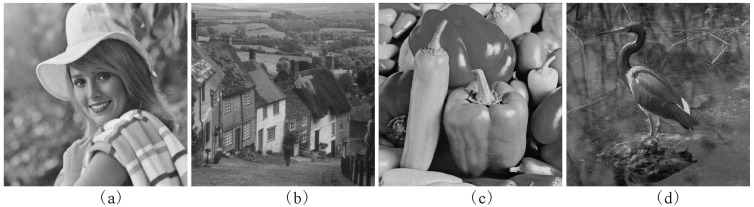
The watermarked images produced by the proposed method. (**a**) Elaine (*PSNR* = 50.72); (**b**) Goldhill (*PSNR* = 50.59); (**c**) Peppers (*PSNR* = 50.53); (**d**) Crane (*PSNR* = 50.62).

The watermarked images produced by the proposed scheme are exhibited in Figure 4. The PSNRs for the watermarked images without attacks are listed in Table 1. As can be seen from Table 1, all the PSNRs of our method are greater than 50db. The high *PSNRs* certify the good imperceptibility of the proposed method. From Table 1, the proposed watermarking scheme outperforms the methods in [2,4,7] in the light of the imperceptibility. Additionally, all of the *NC*s of the watermarks which are extracted from the watermarked images shown in Figure 4 are 1, and the *BER*s of their corresponding reconstructed images are 0, respectively.

#### 5.2.2. Robustness to Attacks

Different kinds of attacks were conducted to verify the robustness of the proposed method. They are Gaussian low-pass filtering (hsize = 5, sigma = 9), Average filtering (5 × 5) Median filtering (7 × 7), Occlusion (25%), Unsharp (alpha = 1), Blurring (Circular average, radius = 3), JPEG compression (Q = 30), Gaussian noise (0.0005), Salt and pepper noise (0.005), Brighten (adds 50 to each pixel of the images), Darken (subtracts 50 from each pixel of the images), Rescaling (1024→512→1024), Rotation (30°→−30°) and Painting, respectively.

**Figure 5:**
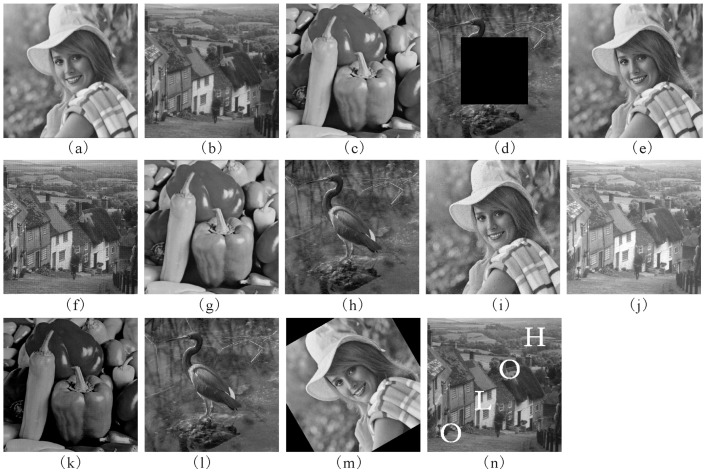
The attacked watermarked images. (**a**) Gaussian low-pass filtering (PSNR = 32.54); (**b**) average filtering (PSNR = 32.42); (**c**) median filtering (PSNR = 34.9); (**d**) occlusion (PSNR = 15.06); (**e**) JPEG (PSNR = 35.95); (**f**) unsharp (PSNR = 27.74); (**g**) burring (PSNR = 34.03); (**h**) Gaussian noise (PSNR = 32.89); (**i**) salt & pepper noise (PSNR = 28.5); (**j**) brighten (PSNR = 14.15); (**k**) darken (PSNR = 14.52); (**l**) rescaling (PSNR = 34.67); (**m**) rotation (PSNR = 13.17); (**n**) painting (PSNR = 20.34).

**Figure 6:**
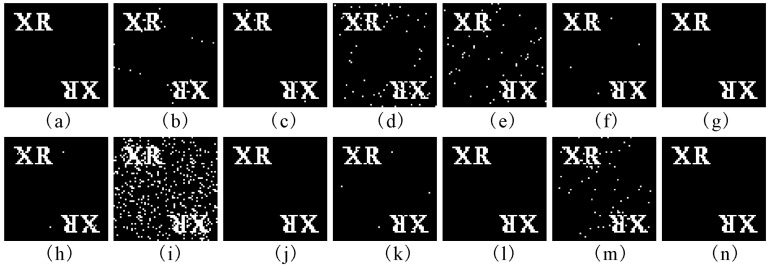
The corresponding reconstructed images of the CGH extracted from the distorted watermarked images in Figure 4. (**a**) Gaussian low-pass filtering; (**b**) average filtering; (**c**) median filtering; (**d**) occlusion; (**e**) JPEG; (**f**) unsharp; (**g**) burring; (**h**) Gaussian noise; (**i**) salt & pepper noise; (**j**) brighten; (**k**) darken; (**l**) rescaling; (**m**) rotation; (**n**) painting.

For all attack cases, a comparison of *NC*s between our method and the schemes from the literature [2,4,7] is listed in Table 2 and Table 3. In Table 4 and Table 5, a comparison of BERs is made between our method and the three schemes. Figure 5 displays some distorted watermarked images of the proposed method together with PSNRs. The corresponding reconstructed images of the mark CGH, which are extracted from the attacked images in Figure 5, are exhibited in Figure 6. As can be seen from Table 2, Table 3, Table 4 and Table 5 and Figure 6, our method has good robustness against various kinds of attack. It can be observed from Table 2 and Table 3 and Figure 6 that most of the *NC*s of the proposed method are above 0.9 and the corresponding reconstructions are clear enough to be recognized. Almost of all BERs of the reconstructed images in Figure 6 are zero, or close to zero. In addition, when the watermarked images are undergone occlusion and rotation attacks, the NCs of the extracted mark hologram are less than 0.8. The reason is that part of the extracted CGH is missing. However, the reconstructed images, such as Figure 6d,m, are clear enough to be recognized because of the characteristic that part of a hologram can still display the whole image [15]. Furthermore, it can be seen from Table 2, Table 3, Table 4 and Table 5 that when the *NC* of the extracted mark CGH is larger than 0.975, the *BER* of its corresponding reconstruction equals to 0. The reason is that the hologram has a strong anti-interference characteristic. Therefore, the robustness of the proposed method can be enhanced by using the hologram as a watermark. From Table 2, Table 3, Table 4 and Table 5, it is apparent that our method is superior to the three algorithms in [2,4,7] under most attacks in terms of NC and BER.

It can be seen from Figure 6i that the quality of the reconstructed image is unsatisfactory when the watermarked image was suffered to the salt and pepper noise attacks. The main causation to this question is analysed as follows. For salt and pepper noise, the image pixel values are altered to 0 or 2*^r^*−1 [28]. Here, *r* is the maximum number of bits that is used in the image. Therefore, after Schur decomposition, the value of the (1,1)^th^ element in *S* matrix of the image block to which the salt and pepper noise is added are changed greatly. It results that the watermarking signal in this damaged block may not be extracted correctly by using Equation (15). As a result, the hologram obtained from the watermarked image under this attack is highly corrupted. Experimental results indicate that the reconstruction of the extracted CGH cannot be distinguished when the noise density of salt and pepper noise is bigger than 0.01.

#### 5.2.3. Key Sensitivity

**Figure 7:**
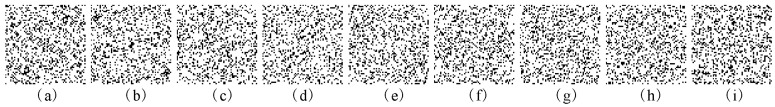
The reconstructed images of the CGHs extracted from watermarked Elaine. (**a**) reconstruction with *SX*(1) = 0.352 + 10^−15^; (**b**) reconstruction with *SY*(1) = 0.865 + 10^−15^; (**c**) reconstruction with *SZ* = 0.752 + 10^−15^; (**d**) reconstruction with *α* = 0.998 + 10^−14^; (**e**) reconstruction with *β* = 3−10^−14^; (**f**) reconstruction with *γ* = 4−10^−15^; (**g**) reconstruction with *t*1 = 3528 + 1; (**h**) reconstruction with *t*2 = 7832 + 1; (**i**) reconstruction with *t*3 = 6832 + 2.

The sensitivity of the reconstructed image of the extracted CGH to slight alterations of the secret keys *SX*(1), *SY*(1), *SZ*, *α*, *β*, *γ*, *t*1, *t*2 and *t*3, is investigated. Figure 7a–i show the decrypted reconstructed images with wrong keys *X*(1) = 0.352 + 10^−15^, *Y*(1) = 0.865 + 10^−15^, *SZ* = 0.752 + 10^−15^, *α* = 0.998 + 10^−14^, *β* = 3−10^−14^, *γ* = 4−10^−15^, *t*1 = 3528 + 1, *t*2 = 7832 + 1 and *t*3 = 6832 + 2, respectively. The *NC*s of the decrypted CGH with the above wrong permutation keys and the *BER*s of the corresponding reconstructions are presented in Table 6. Please note that in the above experiments, the other keys remain correct while a key is varied in decryption. As illustrated in Figure 7a–f, we cannot obtain any information from the decrypted reconstructions visually when the absolute values of deviations of S*X*(1),S*Y*(1), SZ and *γ* are up to 10^−15^ and those of *α* and *β* are up to 10^−14^. In addition, we know from Figure 7g–h that if the parameters *t*_1_ and *t*_2_ are less 1 or more 1 than the right value, the decoded images are noise-like images. Similarly, the decrypted reconstructed image shown in Figure 6i cannot be recognized when the key *t*_3_ are less 2 or more 2 than the correct value. So, the keys *SX*(1), *SY*(1), *SZ*, *α*, *β*, *γ*, *t*1, *t*2 and *t*3 are highly sensitive to the proposed method.

Now we evaluate the key space of the proposed encrypted hologram. In light of the description of the proposed scheme, we know that the key space of the cryptosystem consists of the parameters *SX*(1), *SY*(1), *SZ*, *α*, *β*, *γ*, *t*1, *t*2 and *t*3. Let *KS*_1_, *KS*_2_, *KS*_3_, *KS*_4_, *KS*_5_, *KS*_6_, *KS*_7_, *KS*_8_ and *KS*_9_ be the key spaces of the secret keys *SX*(1), *SY*(1), *SZ*, *α*, *β*, *γ*, *t*1, *t*2 and *t*3, respectively. From Table 6, the parameters *SX*(1), *SY*(1), *SZ*, *α*, *β* and *γ* maintain 15, 15, 15, 14, 14 and 15 digits after the decimal point respectively. So *KS*_1_ × *KS*_2_ × *KS*_3_ × *KS*_4_ × *KS*_5_ × *KS*_6_ = 10^88^. Since 1 ≤ *t*1 ≤ *T* + *PQ*, 1 ≤ *t*2 ≤ *T* + *PQ* and 1 ≤ *t*3 ≤ *T KS*_7_ × *KS*_8_ × *KS*_9_ = (*T* + *PQ*)^2^ × *T*. Since *T* and *PQ* are 10000 and (64 × 64)^2^ in the experiments, *S*_7_ × *S*_8_ × *S*_9_ ≈ 2 × 10^12^. Hence, the total key space of the encryption system is *KS*_1_ × *KS*_2_ × *KS*_3_ × *KS*_4_ × *KS*_5_ × *KS*_6_ × *KS*_7_ × *KS*_8_ × *KS*_9_ ≈ 10^88^ × 2 × 10^12^ ≈ 2^333^. It is clear that the key space of the proposed cryptosystem is far larger than 2^100^ and enormous enough to resist a brute force attack [29]. Therefore, by use of the encrypted hologram, the security level of the proposed watermarking scheme can be improved.

## 6. Conclusions

Based upon the entropy and edge entropy, an adaptive secure image watermarking method that inserts the encrypted hologram into the SFLCT domain is proposed in this paper. Without using the host image, the watermark can be extracted by using the presented method.

A novel chaos-based binary CGH encryption technique which provides a huge key space is developed to fabricate a hologram of a watermark. Compared with the encryption techniques based on conventional optical holography, the advantages of the proposed method are: (1) the parameters of chaotic maps which are used as keys make it easy to save and distribute the keys expediently and safely; (2) the proposed CGH cryptosystem has the advantage in being implemented effectively by the use of a computer. By using the initial values and the parameters of chaotic system as secret keys, the security strength of the watermarking approach is heightened.

In the presented method, the use of entropy and edge entropy helps to choose the suitable embedding positions adaptively for satisfying the invisibility and robustness requirements of the watermarked image. The encrypted hologram watermark is embedded into the SFLCT coefficients with Schur decomposition. The experimental results illustrate that our scheme is not only secure and transparent, but also robust against various kinds of attacks including filtering, JPEG compression, occlusion, unsharp, brighten, darken, blurring, rotation, rescaling and painting attacks, etc.

## Figures and Tables

**Table 1:** The *PSNR*s of the watermarked images without attacks comparing between our method and the schemes in [2,4,7].

	Our Method	Scheme in [7]	Scheme in [2]	Scheme in [4]
Elaine	50.72	45.22	43.15	45.74
Goldhill	50.59	45.71	47.15	49.18
Peppers	50.53	45.4	45.13	47.8
Crane	50.62	45.74	49.11	50.23

**Table 2:** The *NC*s of the mark CGHs which were extracted from the attacked watermarked Elaine and Goldhill comparing between our method and the schemes in [2,4,7].

	Elaine				Goldhill			
Attacks	Our Method	Scheme in [7]	Scheme in [2]	Scheme in [4]	Our Method	Scheme in [7]	Scheme in [2]	Scheme in [4]
Gaussian low-pass filtering	0.994	0.927	0.686	0.681	0.93	0.913	0.613	0.656
Average filtering	0.995	0.925	0.656	0.673	0.929	0.911	0.612	0.65
Median filtering	0.947	0.913	0.637	0.539	0.884	0.77	0.665	0.576
Occlusion	0.824	0.752	0.802	0.821	0.756	0.752	0.806	0.814
Unsharp	0.986	0.94	0.736	0.985	0.941	0.939	0.819	0.984
Blurring	0.99	0.93	0.639	0.806	0.913	0.9	0.601	0.669
JPEG	0.895	0.931	0.646	0.924	0.868	0.927	0.692	0.645
Gaussian noise	0.954	0.97	0.823	0.993	0.948	0.96	0.819	0.922
Salt and pepper noise	0.829	0.813	0.888	0.948	0.855	0.834	0.893	0.938
Brighten	0.817	0.53	0.781	0.797	0.996	0.474	0.987	0.995
Darken	0.992	0.528	0.972	0.991	0.777	0.492	0.847	0.832
Rescaling	0.995	0.795	0.75	0.993	0.952	0.636	0.822	0.966
Rotation	0.8	0.83	0.764	0.777	0.802	0.832	0.768	0.779
Painting	0.986	0.93	0.968	0.983	0.977	0.94	0.973	0.984

**Table 3:** The *NC*s of the mark CGHs which were extracted from the attacked watermarked Peppers and Crane comparing between our method and the schemes in [2,4,7].

	Peppers				Crane			
Attacks	Our Method	Scheme in [7]	Scheme in [2]	Scheme in [4]	Our Method	Scheme in [7]	Scheme in [2]	Scheme in [4]
Gaussian low-pass filtering	0.994	0.895	0.656	0.752	0.97	0.951	0.624	0.615
Average filtering	0.994	0.892	0.655	0.738	0.971	0.95	0.621	0.609
Median filtering	0.971	0.891	0.625	0.501	0.916	0.802	0.653	0.493
Occlusion	0.762	0.761	0.761	0.758	0.78	0.762	0.791	0.8
Unsharp	0.995	0.917	0.636	0.971	0.97	0.957	0.804	0.971
Blurring	0.991	0.866	0.653	0.792	0.965	0.94	0.614	0.628
JPEG	0.883	0.913	0.597	0.644	0.895	0.894	0.786	0.512
Gaussian noise	0.955	0.964	0.715	0.931	0.947	0.961	0.798	0.94
Salt and pepper noise	0.851	0.829	0.827	0.924	0.858	0.841	0.878	0.927
Brighten	0.969	0.528	0.962	0.963	1	0.511	0.985	0.997
Darken	0.886	0.495	0.866	0.877	0.915	0.487	0.95	0.958
Rescaling	0.997	0.781	0.684	0.962	0.988	0.8	0.85	0.976
Rotation	0.832	0.83	0.789	0.785	0.789	0.839	0.759	0.767
Painting	0.97	0.95	0.964	0.955	0.98	0.95	0.972	0.978

**Table 4:** The *BER*s of the reconstructions of the mark CGHs which were extracted from the attacked watermarked Elaine and Goldhill comparing between our method and the schemes in [2,4,7].

	Elaine				Goldhill			
Attacks	Our Method	Scheme in [7]	Scheme in [2]	Scheme in [4]	Our Method	Scheme in [7]	Scheme in [2]	Scheme in [4]
Gaussian low-pass filtering	0	0.007	0.387	0.724	0.005	0.013	0.364	0.407
Average filtering	0	0.008	0.395	0.725	0.005	0.015	0.409	0.405
Median filtering	0.002	0.013	0.412	0.751	0.118	0.33	0.423	0.403
Occlusion	0.023	0.162	0.027	0.026	0.068	0.132	0.042	0.022
JPEG	0.038	0.008	0.395	0.007	0.052	0.01	0.316	0.421
Unsharp	0	0.009	0.12	0	0.003	0.012	0.073	0
Blurring	0	0.006	0.407	0.318	0.008	0.015	0.389	0.391
Gaussian noise	0.001	0.001	0.162	0	0.003	0.003	0.249	0.008
Salt and pepper noise	0.107	0.24	0.078	0.009	0.105	0.208	0.071	0.009
Brighten	0.006	0.401	0.074	0.1	0	0.407	0	0
Darken	0	0.415	0	0	0.103	0.456	0.083	0.097
Rescaling	0	0.295	0.372	0	0.001	0.4	0.327	0.001
Rotation	0.057	0.113	0.104	0.105	0.036	0.131	0.081	0.106
Painting	0	0.065	0.001	0	0	0.011	0	0

**Table 5:** The *BER*s of the reconstructions of the mark CGHs which were extracted from the attacked watermarked Peppers and Crane comparing between our method and the schemes in [2,4,7].

	Peppers				Crane			
Attacks	Our Method	Scheme in [7]	Scheme in [2]	Scheme in [4]	Our method	Scheme in [7]	Scheme in [2]	Scheme in [4]
Gaussian low-pass filtering	0	0.102	0.389	0.34	0.001	0.009	0.624	0.416
Average filtering	0	0.107	0.408	0.363	0.001	0.008	0.621	0.414
Median filtering	0.002	0.108	0.385	0.398	0.005	0.277	0.653	0.415
Occlusion	0.035	0.163	0.058	0.108	0.047	0.155	0.034	0.021
JPEG	0.129	0.008	0.371	0.213	0.113	0.056	0.115	0.342
Unsharp	0	0.009	0.301	0.001	0.001	0.004	0.079	0.001
Blurring	0	0.156	0.408	0.29	0.001	0.014	0.392	0.418
Gaussian noise	0.002	0.002	0.339	0.004	0.002	0.002	0.276	0.005
Salt and pepper noise	0.101	0.241	0.113	0.009	0.104	0.271	0.118	0.006
Brighten	0	0.42	0.002	0.001	0	0.423	0	0
Darken	0.003	0.421	0.003	0.003	0.002	0.417	0.001	0.001
Rescaling	0	0.32	0.377	0.001	0	0.323	0.363	0.001
Rotation	0.043	0.124	0.053	0.067	0.062	0.113	0.103	0.085
Painting	0	0.015	0.001	0.001	0	0.009	0.001	0

**Table 6:** The NC values of the decrypted CGH with the wrong keys and the BER values of the corresponding reconstructions.

	*SX*(1) = 0.352 + 10^−15^	*SY*(1) = 0.865 + 10^−15^	*SZ* = 0.752 + 10^−15^	*α* = 0.998 + 10^−14^	*β* = 3-10^−14^	*γ* = 4-10^−15^	*t*1 = 3528 + 1	*t*2 = 7832 + 1	*t*3 = 6832 + 2
NC	0.506	0.497	0.503	0.514	0.502	0.509	0.509	0.506	0.493
BER	0.755	0.743	0.733	0.756	0.75	0.741	0.743	0.746	0.717
